# Impact of a Hybrid Prevention Program for High School Students on Prescription Drug Misuse Outcomes

**DOI:** 10.3390/bs16010154

**Published:** 2026-01-22

**Authors:** Kenneth W. Griffin, Christopher Williams, Sandra M. Sousa, Gilbert J. Botvin

**Affiliations:** 1 Department of Global and Community Health, George Mason University, Fairfax, VA 22030, USA; 2 National Health Promotion Associates, White Plains, NY 10604, USA; 3 Department of Psychology, School of Natural and Social Sciences, State University of New York at Purchase College, Purchase, NY 10577, USA; 4 Department of Population Health Sciences, Weill Cornell Medical College, New York, NY 10065, USA

**Keywords:** adolescence, prescription drug misuse, prevention, hybrid, e-learning

## Abstract

Prescription drug misuse among youth is a significant public health problem that can lead to negative consequences, including addiction and overdose deaths. This study examined the effectiveness of an evidence-based hybrid approach in preventing prescription drug misuse outcomes in high school students. The prevention program used a combination of e-learning modules and classroom activities to enhance social and personal competence skills and refusal skills to deter prescription drug misuse and other types of substance misuse. Findings indicated that prescription sedative misuse was lower among students who received the hybrid prevention program compared to students in the control group. Perceived risk of using prescription sedatives, painkillers, and stimulants prescribed for someone else was higher in the intervention group relative to the control group students. These findings indicate that a comprehensive, universal school-based hybrid prevention program can produce positive impacts on sedative use and perceived risks of prescription drug misuse.

## 1. Introduction

Prescription drug misuse (PDM) is a critical public health problem in the U.S. and can lead to a variety of negative health effects (McHugh et al., 2015). PDM can have pervasive and detrimental effects on psychological functioning (e.g., depression and anxiety), physical health (e.g., overdose and death) and social functioning (e.g., social isolation, job loss, financial instability) (Ignaszewski, 2021). The far-reaching consequences of PDM, including the opioid epidemic, highlight the urgent need for comprehensive prevention and intervention strategies to address this pressing public health challenge (Bolshakova et al., 2019). Effectively preventing prescription and other forms of substance misuse during adolescence is a critical yet underutilized approach for reducing the morbidity and mortality associated with PDM among adults (Compton et al., 2019). Universal drug prevention programs that aim to reduce the onset and escalation of PDM among youth are needed.

Significant progress has been made in the development and testing of school-based prevention programs that effectively prevent substance misuse during the years of adolescence when the onset of use is highest (Faggiano et al., 2014). Classroom-based approaches that teach social resistance and general life skills are most effective in preventing substance misuse among secondary school students (Botvin & Griffin, 2024). However, implementation of classroom-based prevention programs is often compromised by factors such as insufficient teacher fidelity and the scarcity of classroom time and other resources (Eisman et al., 2024). Hybrid programs that combine e-learning modules for didactic content with classroom time dedicated to discussion and skills practice are effective (Griffin et al., 2022). Previous research has not examined the effects of hybrid universal school-based preventive interventions on PDM. 

The goal of the present study was to examine data from a randomized controlled trial of a hybrid school-based prevention program in reducing PDM behaviors and perceived risks among high school students. We tested an adapted version of the Life Skills Training High School (LST-HS) program, an evidence-based substance use prevention program (Botvin et al., 2015). LST-HS was previously a classroom-only program that focused on alcohol, tobacco, and other drug use, but did not target prescription drug misuse (PDM). The intervention in the present study tested a hybrid adaptation of LST-HS that included new material to address PDM and the perceived risk of harm of PDM. We hypothesized that the hybrid intervention, which used cognitive-behavioral skills-training techniques to enhance social and personal competence skills, would reduce PDM and increase perceived risk of harm associated with PDM at a post-test assessment among intervention youth compared to those who received standard health education.

## 2. Materials and Methods

### 2.1. Procedures

**Figure 1:**
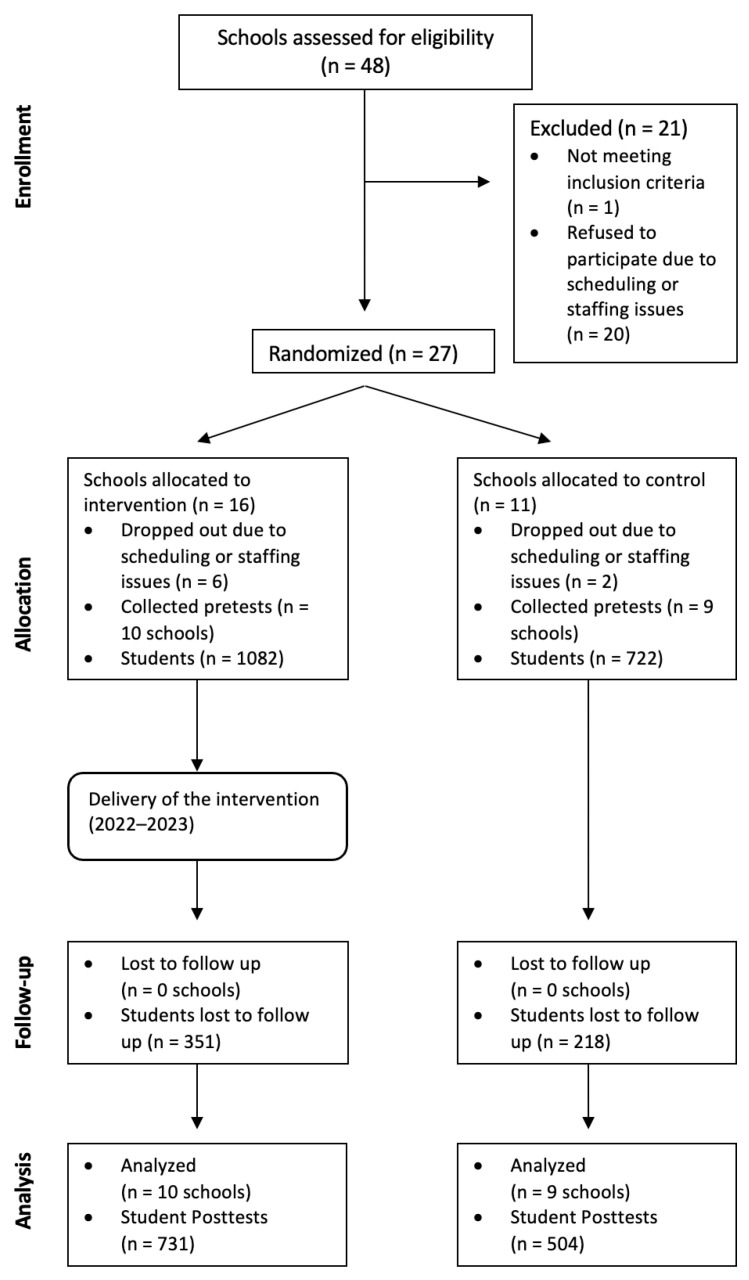
CONSORT (Consolidated Standards of Reporting Trials) diagram.

High schools throughout the U.S. were recruited to participate in this randomized controlled trial via email invitations. Schools were eligible to participate if they provided students with access to web-enabled devices in the classroom or computer lab and provided classroom instruction in English. Randomization occurred at the school level to avoid contamination across conditions. Our original aim was to recruit 30 schools and 3000 students, based on a power analysis (Campbell et al., 2004). However, the COVID-19 pandemic negatively impacted and delayed the planned implementation and data collection schedule for the study. As shown in the CONSORT Diagram (Figure 1), we assessed a larger number of schools (N = 48) for eligibility to account for higher expected dropout rates due to COVID-19. Ultimately, nineteen high schools were randomized and participated in the intervention (N = 10) or control (N = 9) conditions, and the final pre-post analysis sample size was N = 1235. 

Although the original protocol specified 12- and 24-month follow-ups, the 24-month assessment was canceled because the study delays extended the timeline beyond the NIH/NIDA grant performance period. Additionally, widespread school closures and access restrictions resulted in high attrition at the 12-month time point, rendering those data insufficient for meaningful analysis. Consequently, the study analysis focuses on the pre-post timeframe.

The 19 schools that participated in the study were from different regions of the United States, including the Northeast (43.8%), Midwest (31.2%), South (12.5%), and Western states (12.5%). Intervention group youth received the hybrid LST prevention program, which was integrated into the school’s existing health education practices, typically replacing existing prevention programming. Control group youth received existing school health education programming (i.e., “treatment as usual”). We did not collect data concerning the content of existing school health education programming for the control group.

All students in the classroom were eligible to participate in the intervention. However, at the discretion of local school staff, students with significant cognitive impairment or severe learning disabilities were excused from completing the study questionnaire.

### 2.2. Sample

A total of 1235 high school students completed both the pre-test and post-test assessments. This sample was 49% male, and most students were in the 9th grade (44%) or 10th grade (32%). Approximately 30% of students received lunch for free or reduced price, which was a proxy for low socioeconomic background. The racial/ethnic makeup of the sample was White (59%), Black (10%), Asian (3%), or from mixed or other backgrounds (8%), and 25% were Latino/Hispanic. The mean age of the student sample was 15.2 years old. The study protocol and consent procedures were approved by an Institutional Review Board. The protocol is registered at https://clinicaltrials.gov as NCT03219190.

### 2.3. Prevention Program

The hybrid intervention tested in this study was adapted from the Life Skills Training (LST) classroom prevention model, which has been shown to be effective in preventing substance misuse and other risk behaviors among secondary school youth, with findings reported in over 35 scientific peer-reviewed publications (Botvin & Griffin, 2015). The hybrid version consisted of eight e-learning modules and eight teacher-led classroom sessions.

Didactic content was provided in the e-learning modules, which included (a) a personal competence component that taught a variety of self-management skills such as decision-making, coping, goal-setting, and media resistance skills; (b) a social competence component that taught a variety of social skills such as assertiveness, communication, and healthy relationship skills; and (c) a drug resistance component that taught social resistance and refusal skills along with pro-health norms and attitudes. 

Each e-learning module featured teen hosts who introduced lesson topics and led students through immersive, branched scenarios using animated characters and visual storytelling to address developmentally relevant challenges. Interactive knowledge checks provided instant feedback throughout the modules, which concluded with a host-led recap to reinforce the core skills covered. In each class session, the teacher began with a review of key concepts and definitions, followed by facilitated classroom discussions and small-group activities, including role-play and skills practice opportunities. 

The e-learning modules and classroom sessions included new content on PDM that taught participants information, attitudes/norms, and skills designed to prevent the misuse of prescription drugs. Program material emphasized the appropriate and inappropriate use of prescription medications; common myths and realities about prescription drugs; risks of combining prescription drugs with alcohol and other drugs; and resistance skills to deter unwanted offers to misuse prescription drugs. The goal of the present study was to examine intervention effects on the primary outcomes of prescription drug misuse, as well as the perceived risk of PDM, since the intervention was revised to address PDM. Additional secondary outcomes are reported in a separate manuscript (Williams et al., 2025).

### 2.4. Measures

The high school survey assessed demographic variables, including race, ethnicity, and gender, using standard survey items. The frequency of use of sedatives, painkillers, and stimulants without a doctor’s prescription (i.e., prescription drug misuse, PDM) was assessed using three items, adapted from our previous research (Botvin et al., 2001). The items had the stem of “About how often (if ever) do you…” (a) take prescription sedatives without a doctor’s prescription (like Xanax, or Valium)?; (b) take prescription painkillers without a doctor’s prescription (like OxyContin, Vicodin, Percocet, or Codeine)?; and (c) take prescription stimulants without a doctor’s prescription (like Adderall, Ritalin, or Concerta)? Items had a 9-point Likert response scale ranging from 1 = never to 9 = more than once a day. Perceived risk of prescription drug misuse was assessed using three items (α = 0.974) from the National Survey on Drug Use and Health (Substance Abuse and Mental Health Services Administration, 2024), with the stem “How much do you think people risk harming themselves (physically or in other ways), if they “use prescription sedatives (like Xanax, or Valium) regularly, when prescribed for someone else”, “use prescription painkillers (like OxyContin, Vicodin, Percocet, or Codeine) regularly, when prescribed for someone else”, and “use prescription stimulants (like Adderall, Ritalin, or Concerta) regularly, when prescribed for someone else.” Items had a 4-point Likert response scale ranging from 1 = no risk to 4 = great risk, and students were provided with an option to indicate that they were unfamiliar with each of the three prescription medications.

### 2.5. Data Analysis

Bivariate and multivariate statistical methods were used—including *t* tests, chi-squares, and generalized linear models (GLMs)—to analyze data using SPSS Version 31 (IBM Corp., 2025). Pre-test equivalence of the intervention and control group participants was examined, followed by attrition analysis and testing for intervention effects. We tested intervention effects on PDM outcomes using a series of GLMs, in which the pre-test score of the outcome, race/ethnicity, and gender were used as covariates. Robust estimators were specified for the GLM analyses. These estimators relaxed assumptions about the distribution of the dependent variable. For the analyses of the intervention effects, one-tailed significance tests were used due to the unidirectional nature of the hypothesized effects and results of previous research (Griffin et al., 2022). Additional analyses using mixed modeling were conducted to control for the effects of school-level clustering.

## 3. Results

### 3.1. Pre-Test Equivalence

Intervention and control group youth were compared using *t*-tests and chi-square analyses on baseline demographic and behavioral variables. No pre-test differences were observed between the groups on gender distribution or PDM. However, the intervention group consisted of a higher proportion from a racial minority group (vs. White, χ^2^ (1) = 4.61, *p* < 0.05).

### 3.2. Attrition Analysis

The retention rate from the pre-test to post-test assessment was 68.5% and was similar across conditions. There were no differential attrition rates across the intervention and control groups by pre-test substance use or demographic variables.

### 3.3. Intervention Effects

Gender, minority status, and baseline levels of the outcome variables were used as covariates in the models. As shown in Table 1, the post-test adjusted mean for taking sedatives (like Xanax or Valium) without a doctor’s prescription was significantly lower in the intervention group (M = 1.06, SE = 0.02) compared to the control group (M = 1.12, SE = 0.03), Wald χ^2^ (1) = 2.87, *p* < 0.045. The post-test adjusted mean for taking painkillers and stimulants without a doctor’s prescription were each lower in the intervention group (M = 1.11, SE = 0.03, M = 1.09, SE = 0.02, respectively) compared to the control group (M = 1.13, SE = 0.04, M = 1.11, SE = 0.04, respectively) although these differences were not statistically significant. Additional analyses of intervention effects that controlled for school-level clustering were not statistically significant.

As shown in Table 2, the post-test adjusted mean for perceived risk of harm of using prescription sedatives when prescribed for someone else was higher in the intervention students (M = 2.71, SE = 0.02) relative to the control students (M = 2.59, SE = 0.03), Wald χ^2^ (1) = 9.94, *p* < 0.001. The post-test adjusted mean for perceived risk of harm of using prescription painkillers when prescribed for someone else was higher in the intervention students (M = 2.75, SE = 0.02) relative to the control students (M = 2.58, SE = 0.03), Wald χ^2^ (1) = 18.52, *p* < 0.001. The post-test adjusted mean for perceived risk of harm of using prescription stimulants when prescribed for someone else was higher in the intervention students (M = 2.71, SE = 0.02) relative to the control students (M = 2.58, SE = 0.03), Wald χ^2^ (1) = 11.12, *p* < 0.001.

**Figure 2:**
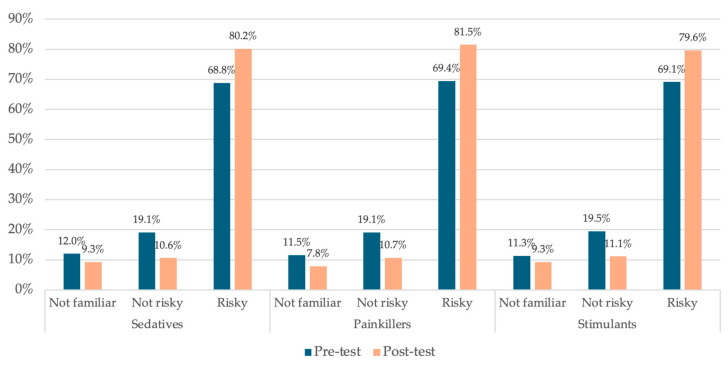
Perceived risk of harm of prescription drug use when prescribed for someone else, Intervention group, Pre-test and post-test assessments.

Figure 2 illustrates how the perceived risk of harm of using prescription medications (when prescribed for someone else) changed from the pre-test to post-test assessments among intervention group students. The proportion of students reporting they were not familiar with sedatives, painkillers, and stimulants each decreased from the pre-test to post-test, ranging from a 17.7% to 32.2% relative reduction. The proportion of students reporting no risk associated with sedatives, painkillers, and stimulants each decreased from the pre-test to post-test, ranging from a 43.1% to 44.5% relative reduction. The proportion of students reporting risk associated with sedatives, painkillers, and stimulants each increased from the pre-test to post-test, ranging from 15.2% to 17.4% relative growth.

## 4. Discussion

The present study examined the effectiveness of a hybrid drug prevention program delivered to students in high school, using a randomized controlled trial. Two to four weeks after the intervention, students who received the hybrid high school program (Life Skills Training) reported lower rates of prescription sedative misuse and greater perceived risk of using prescription medications without a doctor’s prescription, compared to control group students. These findings indicate that an evidence-based, universal school-based prevention program, adapted for hybrid implementation and with new content to prevent PDM, can produce effects on PDM outcomes. The tested hybrid version of LST high school combined brief e-learning modules with in-person class sessions and was found to effectively reduce sedative use behaviors and perceived risk regarding PDM.

The present findings add to the evidence that hybrid substance abuse prevention programs can produce positive outcomes (Griffin et al., 2022). A recent scoping review examined substance use interventions for youth delivered using digital technologies such as mobile phones, video games, or the internet and found that they had substantial potential for increasing access to substance use services among youth, including prevention and treatment (Kwame et al., 2025). Of the 53 studies of digital substance use interventions identified, most were delivered in school settings. However, digital-only intervention approaches may have a relatively limited impact because they fail to provide opportunities for interactivity and group processes—long known to be active ingredients of effective prevention programming according to meta-analytic studies (Tobler & Stratton, 1997). Hybrid prevention models, such as those tested in the present study, that supplement digital delivery of prevention content with classroom activities may offer a more comprehensive prevention approach to youth. They may be more effective because the in-person component facilitates behavioral skills practice and pro-health discussion and peer norm setting that digital-only interventions cannot.

The present study contributes to the literature by showing that an evidence-based substance use prevention program can be expanded to include content related to PDM and produce positive outcomes. Findings from the research on LST demonstrate that a single prevention approach that addresses shared risk and protective factors along with substance-specific content can effectively prevent the use of multiple substances (Botvin & Griffin, 2015). This is an important finding because providing a single comprehensive prevention program is more cost-effective and feasible than providing separate interventions for different types of substances.

Sustained prevention programming across developmental stages is a recognized best practice in school health because it fosters a cumulative effect on student outcomes (Nation et al., 2003). By providing youth with developmentally appropriate prevention curricula, schools can cultivate psychosocial resilience that disrupts the predictable progression from early experimentation with “gateway” substances (e.g., alcohol and tobacco) to more serious drug involvement, including PDM. Intervening consistently throughout the school years reinforces critical refusal skills and maintains protective gains as social pressures and developmental challenges intensify. Spoth et al. (2008, 2013) demonstrated that comprehensive prevention programming delivered in middle school can significantly reduce PDM when youth are followed into late adolescence and young adulthood.

Furthermore, targeting high school youth for PDM is an important strategy because this represents a critical developmental period characterized by escalating risk and increasing independence, coinciding with greater exposure to these medications (Miech et al., 2025). While PDM is generally low among middle school youth, it increases steadily during high school, reaching a peak at age 18 and beyond (McCabe et al., 2019). Therefore, high school is an ideal time for providing students with an intervention designed to prevent PDM, as part of a broader, comprehensive prevention strategy.

Strengths of this study include a rigorous randomized controlled research design, data collected with standardized and confidential self-report instruments, and analysis using multivariate models. Study methods were derived from best practices in prevention science, and the prevention program examined has been shown to be effective in several peer-reviewed journal articles. Limitations include the possible underreporting of sensitive behaviors. While the GLM models tested were robust, they did not control for school-level clustering effects. COVID-19 disrupted our plans for longer-term follow-up. More research is needed to examine the potential sustained effects of the intervention.

## 5. Conclusions

A comprehensive, universal school-based hybrid prevention program produced positive impacts on prescription sedative misuse and the perceived risk of harm of PDM. Future research should investigate the long-term effects of the hybrid prevention model examined in this study, as well as compare the effectiveness of digital-only vs. hybrid prevention models in schools and other settings.

## Figures and Tables

**Table 1:** Adjusted means at post-test for prescription drug misuse, GLMs.

	Intervention Mean (SE)	Control Mean (SE)
Prescription sedative use	1.06 (0.02)	1.12 (0.03) *
Prescription painkiller use	1.11 (0.03)	1.13 (0.04)
Prescription stimulant use	1.09 (0.02)	1.11 (0.04)

* *p* < 0.05.

**Table 2:** Adjusted means at post-test for perceived risk of harm, misusing prescription drugs, GLMs.

	Intervention Mean (SE)	Control Mean (SE)
Prescription sedatives, risk of harm	2.71 (0.02)	2.59 (0.03) **
Prescription painkillers, risk of harm	2.75 (0.02)	2.58 (0.03) **
Prescription stimulants, risk of harm	2.71 (0.02)	2.58 (0.03) **

** *p* < 0.001.

## Data Availability

The raw data supporting the conclusions of this article will be made available by the authors upon reasonable request.
